# A more accurate half-discrete Hardy-Hilbert-type inequality with the logarithmic function

**DOI:** 10.1186/s13660-017-1408-x

**Published:** 2017-06-28

**Authors:** Aizhen Wang, Bicheng Yang

**Affiliations:** Department of Mathematics, Guangdong University of Education, Xingang Zhonglu 351, Guangzhou, 510303 P.R. China

**Keywords:** 26D15, 47A07, Hardy-Hilbert-type inequality, weight function, equivalent form, reverse, operator

## Abstract

By means of the weight functions, the technique of real analysis and Hermite-Hadamard’s inequality, a more accurate half-discrete Hardy-Hilbert-type inequality related to the kernel of logarithmic function and a best possible constant factor is given. Moreover, the equivalent forms, the operator expressions, the reverses and some particular cases are also considered.

## Introduction

Assuming that $p>1$, $\frac{1}{p}+\frac{1}{q}=1$, $f(x),g(y)\geq0$, $f\in L^{p}( \mathbf{R}_{+})$, $g\in L^{q}(\mathbf{R}_{+})$, $\Vert f \Vert _{p} = (\int_{0}^{\infty }f^{p}(x)\,dx)^{\frac{1}{p}}>0$, $\Vert g \Vert _{q}>0$, we have the following Hardy-Hilbert integral inequality (*cf.* [1]):

1 $$ \int_{0}^{\infty} \int_{0}^{\infty}\frac{f(x)g(y)}{x+y}\,dx\,dy< \frac{\pi }{\sin(\pi/p)} \Vert f \Vert _{p} \Vert g \Vert _{q}, $$

where the constant factor $\frac{\pi}{\sin(\pi/p)}$ is the best possible. If $a_{m},b_{n}\geq0$, $a=\{a_{m}\}_{m=1}^{\infty}\in l^{p}$, $b=\{b_{n}\}_{n=1}^{\infty}\in l^{q}$, $\Vert a \Vert _{p}=(\sum_{m=1}^{\infty }a_{m}^{p})^{\frac{1}{p}}>0$, $\Vert b \Vert _{q}>0$, then we have the following Hardy-Hilbert inequality with the same best possible constant factor $\frac{\pi}{\sin(\pi/p)}$ (*cf.* [1]):

2 $$ \sum_{m=1}^{\infty}\sum _{n=1}^{\infty}\frac{a_{m}b_{n}}{m+n}< \frac {\pi}{\sin(\pi/p)} \Vert a \Vert _{p} \Vert b \Vert _{q}. $$

Inequalities (1) and (2) are important in analysis and its applications (*cf.* [1–3]).

Suppose that $\mu_{i},\nu_{j}>0$ ($i,j\in\mathbf{N}=\{1,2,\ldots \}$),

3 $$ U_{m}:=\sum_{i=1}^{m} \mu_{i},\quad\quad V_{n}:=\sum_{j=1}^{n} \nu_{j} \quad (m,n\in \mathbf{N}). $$

We have the following Hardy-Hilbert-type inequality (*cf.* Theorem 321 of [1], replacing $\mu_{m}^{1/q}a_{m}$ and $\nu_{n}^{1/p}b_{n}$ by $a_{m}$ and $b_{n}$):

4 $$ \sum_{m=1}^{\infty}\sum _{n=1}^{\infty}\frac {a_{m}b_{n}}{U_{m}+V_{n}}< \frac{\pi}{\sin(\frac{\pi}{p})} \Biggl( \sum_{m=1}^{\infty}\frac {a_{m}^{p}}{\mu _{m}^{p-1}} \Biggr) ^{\frac{1}{p}} \Biggl( \sum_{n=1}^{\infty} \frac {b_{n}^{q}}{\nu_{n}^{q-1}} \Biggr) ^{\frac{1}{q}}. $$

For $\mu_{i}=\nu_{j}=1$ ($i,j\in\mathbf{N}$), (4) reduces to (2).

In 1998, by introducing an independent parameter $\lambda\in(0,1]$, Yang [4] gave an extension of (1) with the kernel $\frac{1}{(x+y)^{\lambda}}$ for $p=q=2$. Recently, Yang [3] gave some extensions of (1) and (2) as follows: If $\lambda _{1},\lambda_{2}\in\mathbf{R}$, $\lambda_{1}+\lambda_{2}=\lambda $, $k_{\lambda}(x,y)$ is a non-negative homogeneous function of degree −*λ*, with $k(\lambda_{1})=\int_{0}^{\infty}k_{\lambda }(t,1)t^{\lambda_{1}-1}\,dt\in\mathbf{R}_{+}$, $\phi(x)=x^{p(1-\lambda _{1})-1}$, $\psi(x)=x^{q(1-\lambda_{2})-1}$, $f(x), g(y)\geq0$,

$$f\in L_{p,\phi}(\mathbf{R}_{+})= \biggl\{ f; \Vert f \Vert _{p,\phi }:= \biggl( \int_{0}^{\infty}\phi(x) \bigl\vert f(x) \bigr\vert ^{p}\,dx \biggr)^{\frac{1}{p}}< \infty \biggr\} , $$

$g\in L_{q,\psi}(\mathbf{R}_{+})$, $\Vert f \Vert _{p,\phi }, \Vert g \Vert _{q,\psi}>0$, then we have

5 $$ \int_{0}^{\infty} \int_{0}^{\infty}k_{\lambda }(x,y)f(x)g(y)\,dx\,dy< k( \lambda _{1}) \Vert f \Vert _{p,\phi} \Vert g \Vert _{q,\psi }, $$

where the constant factor $k(\lambda_{1})$ is the best possible. Moreover, if $k_{\lambda}(x,y)$ keeps a finite value and $k_{\lambda }(x,y)x^{\lambda _{1}-1} (k_{\lambda}(x,y)y^{\lambda_{2}-1})$ is decreasing with respect to $x>0$ ($y>0$), then, for $a_{m,}b_{n}\geq0$,

$$a\in l_{p,\phi}= \Biggl\{ a; \Vert a \Vert _{p,\phi}:= \Biggl( \sum_{n=1}^{\infty}\phi (n) \vert a_{n} \vert ^{p} \Biggr)^{\frac{1}{p}}< \infty \Biggr\} , $$

$b=\{b_{n}\}_{n=1}^{\infty}\in l_{q,\psi}$, $\Vert a \Vert _{p,\phi}, \Vert b \Vert _{q,\psi }>0$, we have the following inequality with the same best possible constant factor $k(\lambda_{1})$:

6 $$ \sum_{m=1}^{\infty}\sum _{n=1}^{\infty}k_{\lambda }(m,n)a_{m}b_{n}< k( \lambda_{1}) \Vert a \Vert _{p,\phi} \Vert b \Vert _{q,\psi}. $$

In 2015, Yang [5] gave an extension of (6) for the kernel $k_{\lambda}(m,n)=\frac{1}{(m+n)^{\lambda}}$ and (4) as follows:

7 $$\begin{aligned}& \sum_{m=1}^{\infty}\sum _{n=1}^{\infty}\frac{a_{m}b_{n}}{(U_{m}+V_{n})^{\lambda}} \\& \quad < B(\lambda_{1},\lambda_{2}) \Biggl[ \sum _{m=1}^{\infty}\frac{U_{m}^{p(1-\lambda_{1})-1}a_{m}^{p}}{\mu_{m}^{p-1}} \Biggr] ^{\frac {1}{p}} \Biggl[ \sum_{n=1}^{\infty} \frac{V_{n}^{q(1-\lambda _{2})-1}b_{n}^{q}}{\nu _{n}^{q-1}} \Biggr] ^{\frac{1}{q}}, \end{aligned}$$

where the constant $B(\lambda_{1},\lambda_{2})$ is the best possible, and $B(u,v)$ ($u,v>0$) is the beta function. Some other results including multidimensional Hilbert-type inequalities are provided by [6–24].

About half-discrete Hilbert-type inequalities with the non-homogeneous kernels, Hardy *et al.* provided a few results in Theorem 351 of [1]. Yang [25] gave an inequality with the kernel $\frac {1}{(1+nx)^{\lambda}}$ by introducing an interval variable and proved that the constant factor is the best possible. Zhong *et al.* [26–28] investigated a few half-discrete Hilbert-type inequalities with the particular kernels. Applying the weight functions, a half-discrete Hilbert-type inequality with a general homogeneous kernel of degree $-\lambda\in\mathbf{R}$ and a best constant factor $k ( \lambda_{1} ) $ is proved as follows (*cf.* [29]):

8 $$ \int_{0}^{\infty}f(x)\sum_{n=1}^{\infty}k_{\lambda }(x,n)a_{n}\,dx< k( \lambda _{1}) \Vert f \Vert _{p,\phi} \Vert a \Vert _{q,\psi }. $$

A half-discrete Hilbert-type inequality with a general non-homogeneous kernel and a best constant factor is given by Yang [30].

In this paper, by means of the weight functions, the technique of real analysis and Hermite-Hadamard’s inequality, a more accurate half-discrete Hardy-Hilbert-type inequality related to the kernel of logarithmic function and a best possible constant factor is given, which is an extension of (8) in a particular kernel of degree 0 similar to (7). The equivalent forms, the operator expressions, the equivalent reverses and some particular cases are also considered.

## Some lemmas

In the following, we agree that $\nu_{j}>0$ ($j\in\mathbf{N}$), $V_{n}:=\sum_{j=1}^{n}\nu_{j}$, $\mu(t)$ is a positive continuous function in $\mathbf{R}_{+}=(0,\infty)$,

$$U(x):= \int_{0}^{x}\mu(t)\,dt< \infty\quad \bigl(x\in [0,\infty)\bigr), $$

$0\leq\widetilde{\nu}_{n}\leq\frac{\nu_{n}}{2}$, $\widetilde {V}_{n}=V_{n}-\widetilde{\nu}_{n}$, $\nu(t):=\nu_{n}$, $t\in(n-\frac{1}{2},n+\frac{1}{2} ]$ ($n\in\mathbf{N}$), and

$$V(y):= \int_{\frac{1}{2}}^{y}\nu(t)\,dt \quad \biggl(y \in \bigg[\frac {1}{2},\infty \bigg)\biggr), $$

$p\neq0,1$, $\frac{1}{p}+\frac{1}{q}=1$, $0<\sigma<\gamma$, $\sigma\leq 1$, $\delta\in\{-1,1\}$, $f(x),a_{n}\geq0$ ($x\in\mathbf{R}_{+}$, $n\in \mathbf{N}$), $\Vert f \Vert _{p,\Phi_{\delta}}=(\int _{0}^{\infty}\Phi_{\delta }(x)f^{p}(x)\,dx)^{\frac{1}{p}}$, $\Vert a \Vert _{q,\widetilde {\Psi}}=(\sum_{n=1}^{\infty}\widetilde{\Psi}(n)b_{n}^{q})^{\frac{1}{q}}$, where

$$\Phi_{\delta}(x):=\frac{U^{p(1-\delta\sigma)-1}(x)}{\mu^{p-1}(x)},\quad\quad \widetilde{\Psi}(n):= \frac{\widetilde{V}_{n}^{q(1-\sigma)-1}}{\nu _{n}^{q-1}} \quad (x\in\mathbf{R}_{+},n\in\mathbf{N}). $$

### Example 1

For $\rho>0$, we set

$$h(t):=\ln\biggl(1+\frac{\rho}{t^{\gamma}}\biggr) \quad (t\in\mathbf{R}_{+}). $$

(i) Setting $u=\rho t^{-\gamma}$, we find

9 $$\begin{aligned} k(\sigma) :=& \int_{0}^{\infty}t^{\sigma-1}\ln\biggl(1+ \frac{\rho }{t^{\gamma}}\biggr)\,dt \\ =&\frac{\rho^{\sigma/\gamma}}{\gamma} \int_{0}^{\infty}u^{\frac {-\sigma }{\gamma}-1}\ln(1+u)\,du= \frac{-\rho^{\sigma/\gamma}}{\sigma}\int_{0}^{\infty}\ln(1+u)\,du^{\frac{-\sigma}{\gamma}} \\ =&-\frac{\rho^{\sigma/\gamma}}{\sigma} \biggl[ u^{\frac{-\sigma }{\gamma}}\ln(1+u)|_{0}^{\infty}- \int_{0}^{\infty}\frac{u^{\frac{-\sigma }{\gamma}}}{1+u}\,du \biggr] \\ =&\frac{\rho^{\sigma/\gamma}}{\sigma} \int_{0}^{\infty}\frac {u^{-\frac{\sigma}{\gamma}}}{1+u}\,du= \frac{\rho^{\sigma/\gamma}\pi}{\sigma \sin\pi (1- \frac{ \sigma}{\gamma})}=\frac{\rho^{\sigma/\gamma}\pi}{\sigma \sin(\frac{\pi\sigma}{\gamma})}. \end{aligned}$$

(ii) We obtain, for $t>0$, $h(t)=\ln(1+\frac{\rho}{t^{\gamma}})>0$,

$$\frac{d}{dt}h(t)=\frac{-\rho\gamma}{(t^{\gamma}+\rho)t}< 0,\quad\quad \frac {d^{2}}{dt^{2}}h(t)>0. $$

It is evident that, for $\sigma\leq1$, $t^{\sigma-1}h(t)>0$, we have

$$\frac{d}{dt}\bigl(t^{\sigma-1}h(t)\bigr)< 0,\quad\quad \frac{d^{2}}{dt^{2}} \bigl(t^{\sigma-1}h(t)\bigr)>0. $$

(iii) Since for $n\in\mathbf{N}$, $V(y)>0$, $V^{\prime}(y)=\nu _{n}>0$, $V^{\prime \prime}(y)=0$ ($y\in(n-\frac{1}{2},n+\frac{1}{2})$), then, for $c>0$, we have

$$\begin{aligned}& h\bigl(cV(y)\bigr)V^{\sigma-1}(y) > 0,\quad\quad \frac{d}{dy}\bigl(h\bigl(cV(y) \bigr)V^{\sigma-1}(y)\bigr)< 0, \\& \frac{d^{2}}{dy^{2}}\bigl(h\bigl(cV(y)\bigr)V^{\sigma-1}(y)\bigr) > 0\quad \biggl(y\in\biggl(n-\frac {1}{2},n+\frac{1}{2}\biggr) \biggr). \end{aligned}$$

### Lemma 1

*If* $g(t)>0$, $g^{\prime}(t)<0$, $g^{\prime\prime}(t)>0$ ($t\in (\frac{1}{2},\infty)$), *satisfying* $\int_{\frac{1}{2} }^{\infty}g(t)\,dt\in\mathbf{R}_{+}$, *then we have*

10 $$ \int_{1}^{\infty}g(t)\,dt< \sum _{n=1}^{\infty}g(n)< \int_{\frac {1}{2}}^{\infty }g(t)\,dt. $$

### Proof

For $n_{0}\in\mathbf{N}\backslash\{1\}$, by the assumptions and Hermite-Hadamard’s inequality (*cf.* [31]), we have

11 $$ \int_{n}^{n+1}g(t)\,dt< g(n)< \int_{n-\frac{1}{2}}^{n+\frac{1}{2}}g(t)\,dt \quad (n=1,\ldots,n_{0}). $$

It follows that

$$0< \int_{1}^{n_{0}+1}g(t)\,dt< \sum _{n=1}^{n_{0}}g(n)< \sum_{n=1}^{n_{0}} \int _{n-\frac{1}{2}}^{n+\frac{1}{2}}g(t)\,dt= \int_{\frac{1}{2}}^{n_{0}+\frac{1}{2} }g(t)\,dt< \infty. $$

In the same way, we still have

$$0< \int_{n_{0}+1}^{\infty}g(t)\,dt\leq\sum _{n=n_{0}+1}^{\infty}g(n)\leq \int_{n_{0}+\frac{1}{2}}^{\infty}g(t)\,dt< \infty. $$

Hence, adding the above two inequalities, we have (10). The lemma is proved. □

### Lemma 2

*Assuming that* $\rho>0$, *we define the following weight functions*:

12 $$\begin{aligned}& \omega_{\delta}(\sigma,x) : =\sum_{n=1}^{\infty} \frac{U^{\delta \sigma }(x)\nu_{n}}{\widetilde{V}_{n}^{1-\sigma}}\ln \biggl[ 1+\frac{\rho}{(U^{\delta}(x)\widetilde{V}_{n})^{\gamma}} \biggr] ,\quad x\in \mathbf{R}_{+}, \end{aligned}$$

13 $$\begin{aligned}& \varpi_{\delta}(\sigma,n) : = \int_{0}^{\infty}\frac{\widetilde{V}_{n}^{\sigma}\mu(x)}{U^{1-\delta\sigma}(x)}\ln \biggl[ 1+ \frac{\rho }{(U^{\delta}(x)\widetilde{V}_{n})^{\gamma}} \biggr] \,dx,\quad n\in\mathbf{N}. \end{aligned}$$

*Then we have the following inequalities*:

14 $$\begin{aligned}& \omega_{\delta}(\sigma,x) < k(\sigma) \quad (x\in\mathbf{R}_{+}), \end{aligned}$$

15 $$\begin{aligned}& \varpi_{\delta}(\sigma,n) \leq k(\sigma) \quad (n\in\mathbf{N}), \end{aligned}$$

*where* $k(\sigma)$ *is determined by* (9).

### Proof

Since

16 $$\begin{aligned} V(n) =& \int_{\frac{1}{2}}^{n+\frac{1}{2}}\nu(t)\,dt-\frac{\nu _{n}}{2}=V_{n}-\frac{\nu_{n}}{2} \\ \leq&\widetilde{V}_{n}\leq V_{n}=V\biggl(n+ \frac{1}{2}\biggr), \end{aligned}$$

and for $t\in(n-\frac{1}{2},n+\frac{1}{2})$, $V^{\prime}(t)=\nu_{n}$, by Examples 1(ii)-(iii), (16), (11) and (10), we have

$$\begin{aligned}& \frac{U^{\delta\sigma}(x)\nu_{n}}{\widetilde{V}_{n}^{1-\sigma }}\ln \biggl[ 1+\frac{\rho}{(U^{\delta}(x)\widetilde{V}_{n})^{\gamma}} \biggr] \\& \quad \leq \frac{U^{\delta\sigma}(x)\nu_{n}}{V^{1-\sigma}(n)}\ln \biggl[ 1+\frac{\rho}{(U^{\delta}(x)V(n))^{\gamma}} \biggr] \\& \quad < \int_{n-\frac{1}{2}}^{n+\frac{1}{2}}\frac{U^{\delta\sigma }(x)V^{\prime }(t)}{V^{1-\sigma}(t)}\ln \biggl[ 1+ \frac{\rho}{(U^{\delta }(x)V(t))^{\gamma}} \biggr] \,dt \quad (n\in\mathbf{N}), \\& \begin{aligned} \omega_{\delta}(\sigma,x) &< \sum_{n=1}^{\infty} \int_{n-\frac {1}{2}}^{n+\frac{1}{2}}\frac{U^{\delta\sigma}(x)V^{\prime}(t)}{V^{1-\sigma }(t)}\ln \biggl[ 1+\frac{\rho}{(U^{\delta}(x)V(t))^{\gamma}} \biggr] \,dt \\ &= \int_{\frac{1}{2}}^{\infty}\frac{U^{\delta\sigma}(x)V^{\prime }(t)}{V^{1-\sigma}(t)}\ln \biggl[ 1+ \frac{\rho}{(U^{\delta}(x)V(t))^{\gamma }} \biggr] \,dt. \end{aligned} \end{aligned}$$

Setting $u=U^{\delta}(x)V(t)$ in the above, by (9), we find

$$\begin{aligned} \omega_{\delta}(\sigma,x) < & \int_{0}^{U^{\delta}(x)V(\infty)}\ln \biggl(1+\frac{\rho}{u^{\gamma}}\biggr)\frac{U^{\delta\sigma}(x)U^{-\delta}(x)}{(uU^{-\delta}(x))^{1-\sigma}}\,du \\ \leq& \int_{0}^{\infty}u^{\sigma-1}\ln\biggl(1+ \frac{\rho}{u^{\gamma}}\biggr)\,du=k(\sigma). \end{aligned}$$

Hence, (14) follows.

Setting $u=\widetilde{V}_{n}U^{\delta}(x)$ in (13), we find $du=\delta\widetilde{V}_{n}U^{\delta-1}(x)\mu(x)\,dx$ and

$$\begin{aligned} \varpi_{\delta}(\sigma,n) =&\frac{1}{\delta} \int_{\widetilde{V}_{n}U^{\delta}(0)}^{\widetilde{V}_{n}U^{\delta}(\infty)}\frac {\widetilde{V}_{n}^{\sigma}\widetilde{V}_{n}^{-1}(\widetilde{V}_{n}^{-1}u)^{\frac {1}{\delta}-1}}{(\widetilde{V}_{n}^{-1}u)^{\frac{1}{\delta}-\sigma}}\ln \biggl(1+\frac{\rho}{u^{\gamma}}\biggr)\,du \\ =&\frac{1}{\delta} \int_{\widetilde{V}_{n}U^{\delta}(0)}^{\widetilde {V}_{n}U^{\delta}(\infty)}u^{\sigma-1}\ln\biggl(1+ \frac{\rho}{u^{\gamma}}\biggr)\,du. \end{aligned}$$

If $\delta=1$, then

$$\varpi_{1}(\sigma,n)= \int_{0}^{\widetilde{V}_{n}U(\infty)}u^{\sigma -1}\ln\biggl(1+ \frac{\rho}{u^{\gamma}}\biggr)\,du\leq \int_{0}^{\infty}u^{\sigma -1}\ln\biggl(1+ \frac{\rho}{u^{\gamma}}\biggr)\,du; $$

if $\delta=-1$, then

$$\varpi_{-1}(\sigma,n)=- \int_{\infty}^{\widetilde{V}_{n}/U(\infty )}u^{\sigma-1}\ln\biggl(1+ \frac{\rho}{u^{\gamma}}\biggr)\,du\leq \int_{0}^{\infty }u^{\sigma-1}\ln\biggl(1+ \frac{\rho}{u^{\gamma}}\biggr)\,du. $$

Then by (9), we have (15). The lemma is proved. □

### Note

If $U(\infty)=\infty$, then (15) keeps the form of an equality.

### Lemma 3

*If* $\rho>0$, *there exists a* $n_{0}\in\mathbf{N}$, *such that* $\nu_{n}\geq\nu_{n+1}$ ($n\in \{n_{0},n_{0}+1,\ldots\}$), *and* $V_{\infty}=\infty$, *then*: (i)* for* $x\in \mathbf{R}_{+}$, *we have*

17 $$ k(\sigma) \bigl(1-\theta_{\delta}(\sigma,x)\bigr)< \omega_{\delta}( \sigma,x), $$

*where*

18 $$\begin{aligned} \theta_{\delta}(\sigma,x) :=&\frac{1}{k(\sigma)} \int_{0}^{U^{\delta }(x)V_{n_{0}}}u^{\sigma-1}\ln\biggl(1+ \frac{\rho}{u^{\gamma}}\biggr)\,du \\ =&O\bigl(\bigl(U(x)\bigr)^{\frac{\delta\sigma}{2}}\bigr)\in(0,1); \end{aligned}$$

(ii) *for any* $b>0$, *we have*

19 $$ \sum_{n=1}^{\infty}\frac{\nu_{n}}{\widetilde{V}_{n}^{1+b}}= \frac{1}{b} \biggl( \frac{1}{V _{n_{0}}^{b}}+bO(1) \biggr) . $$

### Proof

(i) Since for $t\in(n,n+1)$ ($n\geq n_{0}$), $\nu _{n}\geq \nu_{n+1}=V^{\prime}(t+\frac{1}{2})$, by Examples 1(iii) and (11), we have

$$\begin{aligned} \omega_{\delta}(\sigma,x) \geq&\sum_{n=n_{0}}^{\infty} \ln \biggl[ 1+\frac{\rho}{(U^{\delta}(x)V_{n})^{\gamma}} \biggr] \frac{U^{\delta \sigma }(x)\nu_{n}}{V_{n}^{1-\sigma}} \\ =&\sum_{n=n_{0}}^{\infty}\ln \biggl[ 1+ \frac{\rho}{(U^{\delta}(x)V(n+ \frac{1}{2}))^{\gamma}} \biggr] \frac{U^{\delta\sigma}(x)\nu_{n}}{V^{1-\sigma}(n+\frac{1}{2})} \\ >&\sum_{n=n_{0}}^{\infty} \int_{n}^{n+1}\ln \biggl[ 1+\frac{\rho}{(U^{\delta}(x)V(t+\frac{1}{2}))^{\gamma}} \biggr] \frac{U^{\delta \sigma }(x)V^{\prime}(t+\frac{1}{2})\,dt}{V^{1-\sigma}(t+\frac{1}{2})} \\ =& \int_{n_{0}}^{\infty}\ln \biggl[ 1+\frac{\rho}{(U^{\delta }(x)V(t+\frac{1}{2}))^{\gamma}} \biggr] \frac{U^{\delta\sigma}(x)V^{\prime}(t+\frac {1}{2})\,dt}{V^{1-\sigma}(t+\frac{1}{2})}. \end{aligned}$$

Setting $u=U^{\delta}(x)V(t+\frac{1}{2})$ in the above, in view of $V_{\infty}=\infty$, by (9), we find

$$\begin{aligned}& \begin{aligned} \omega_{\delta}(\sigma,x) &> \int_{U^{\delta}(x)V(n_{0}+\frac{1}{2})}^{\infty}u^{\sigma-1}\ln\biggl(1+ \frac{\rho}{u^{\gamma}}\biggr)\,du \\ &=k(\sigma)- \int_{0}^{U^{\delta}(x)V_{n_{0}}}u^{\sigma-1}\ln \biggl(1+ \frac{\rho}{u^{\gamma}}\biggr)\,du \\ &=k(\sigma) \bigl(1-\theta_{\delta}(\sigma,x)\bigr), \end{aligned} \\& \theta_{\delta}(\sigma,x) =\frac{1}{k(\sigma)} \int_{0}^{U^{\delta }(x)V_{n_{0}}}u^{\sigma-1}\ln\biggl(1+ \frac{\rho}{u^{\gamma}}\biggr)\,du\in(0,1). \end{aligned}$$

Since $F(u)=u^{\frac{\sigma}{2}}\ln(1+\frac{\rho}{u^{\gamma}})$ is continuous in $(0,\infty)$ satisfying $F(u)\rightarrow0$ ($u\rightarrow 0^{+}$ or $u\rightarrow\infty$), there exists a constant $L>0$, such that $F(u)\leq L$, namely,

$$\ln\biggl(1+\frac{\rho}{u^{\gamma}}\biggr)\leq Lu^{\frac{-\sigma}{2}} \quad \bigl(u\in (0, \infty)\bigr). $$

Hence we find

$$0< \theta_{\delta}(\sigma,x)\leq\frac{L}{k(\sigma)} \int _{0}^{U^{\delta }(x)V_{n_{0}}}u^{\frac{\sigma}{2}-1}\,du= \frac{2L(U^{\delta }(x)V_{n_{0}})^{\sigma/2}}{k(\sigma)\sigma}, $$

and then (18) follows.

(ii) For $b>0$, by (11), we find

$$\begin{aligned}& \begin{aligned} \sum_{n=1}^{\infty}\frac{\nu_{n}}{\widetilde{V}_{n}^{1+b}} &\leq \sum_{n=1}^{n_{0}}\frac{\nu_{n}}{\widetilde{V}_{n}^{1+b}}+ \sum_{n=n_{0}+1}^{\infty}\frac{\nu_{n}}{V^{1+b}(n)} \\ &\leq\sum_{n=1}^{n_{0}}\frac{\nu_{n}}{\widetilde{V}_{n}^{1+b}}+ \sum_{n=n_{0}+1}^{\infty} \int_{n-\frac{1}{2}}^{n+\frac{1}{2}}\frac{V^{\prime}(t)}{V^{1+b}(t)}\,dt \\ &=\sum_{n=1}^{n_{0}}\frac{\nu_{n}}{\widetilde{V}_{n}^{1+b}}+ \int _{n_{0}+\frac{1}{2}}^{\infty}\frac{dV(t)}{V^{1+b}(t)} \\ &=\frac{1}{b} \Biggl( \frac{1}{V_{n_{0}}^{b}}+b\sum _{n=1}^{n_{0}}\frac {\nu _{n}}{\widetilde{V}_{n}^{1+b}} \Biggr) ; \end{aligned} \\& \begin{aligned} \sum_{n=1}^{\infty}\frac{\nu_{n}}{\widetilde{V}_{n}^{1+b}} &\geq \sum_{n=n_{0}}^{\infty}\frac{\nu_{n+1}}{V^{1+b}(n+\frac{1}{2})}\geq \sum _{n=n_{0}}^{\infty} \int_{n}^{n+1}\frac{V^{\prime}(t+\frac{1}{2})}{ V^{1+b}(t+\frac{1}{2})}\,dt \\ &= \int_{n_{0}}^{\infty}\frac{dV(t+\frac{1}{2})}{V^{1+b}(t+\frac {1}{2})}=\frac{1}{bV^{b}(n_{0}+\frac{1}{2})}=\frac{1}{bV_{n_{0}}^{b}}. \end{aligned} \end{aligned}$$

Hence we have (19). The lemma is proved. □

### Note

For example, $\nu_{n}=\frac{1}{n^{\beta}}$ ($n\in \mathbf{N}$; $0\leq\beta\leq1$) satisfies the conditions of Lemma 3 (for $n_{0}=1$).

## Main results and operator expressions

### Theorem 1

*If* $\rho>0$, $k(\sigma)$ *is determined by* (9), *then*, *for* $p>1$, $0< \Vert f \Vert _{p,\Phi _{\delta}}$, $\Vert a \Vert _{q,\widetilde{\Psi}}<\infty$, *we have the following equivalent inequalities*:

20 $$\begin{aligned}& I:=\sum_{n=1}^{\infty} \int_{0}^{\infty}\ln \biggl[ 1+\frac{\rho}{(U^{\delta}(x)\widetilde{V}_{n})^{\gamma}} \biggr] a_{n}f(x)\,dx< k(\sigma ) \Vert f \Vert _{p,\Phi_{\delta}} \Vert a \Vert _{q,\widetilde{\Psi}}, \end{aligned}$$

21 $$\begin{aligned}& \begin{aligned}[b] J_{1} &:= \Biggl\{ \sum_{n=1}^{\infty} \frac{\nu_{n}}{\widetilde{V}_{n}^{1-p\sigma}} \biggl[ \int_{0}^{\infty}\ln \biggl( 1+\frac{\rho}{(U^{\delta}(x)\widetilde{V}_{n})^{\gamma}} \biggr) f(x)\,dx \biggr] ^{p} \Biggr\} ^{\frac{1}{p}} \\ &< k(\sigma) \Vert f \Vert _{p,\Phi_{\delta}}, \end{aligned} \end{aligned}$$

22 $$\begin{aligned}& \begin{aligned}[b] J_{2} &:= \Biggl\{ \int_{0}^{\infty}\frac{\mu(x)}{U^{1-q\delta\sigma }(x)} \Biggl[ \sum_{n=1}^{\infty}\ln \biggl( 1+ \frac{\rho}{(U^{\delta}(x)\widetilde{V}_{n})^{\gamma}} \biggr) a_{n} \Biggr] ^{q}\,dx \Biggr\} ^{\frac {1}{q}} \\ &< k(\sigma) \Vert a \Vert _{q,\widetilde{\Psi}}. \end{aligned} \end{aligned}$$

### Proof

By Hölder’s inequality with weight (*cf.* [31]), we have

23 $$\begin{aligned}& \biggl\{ \int_{0}^{\infty}\ln \biggl[ 1+\frac{\rho}{(U^{\delta}(x)\widetilde{V}_{n})^{\gamma}} \biggr] f(x)\,dx \biggr\} ^{p} \\& \quad = \biggl\{ \int_{0}^{\infty}\ln \biggl[ 1+\frac{\rho}{(U^{\delta}(x)\widetilde{V}_{n})^{\gamma}} \biggr] \biggl[ \frac{U^{\frac{1-\delta \sigma}{q}}(x)f(x)}{\widetilde{V}_{n}^{\frac{1-\sigma}{p}}\mu^{\frac {1}{q}}(x)} \biggr] \biggl[ \frac{\widetilde{V}_{n}^{\frac{1-\sigma}{p}}\mu^{\frac {1}{q}}(x)}{U^{\frac{1-\delta\sigma}{q}}(x)} \biggr] \,dx \biggr\} ^{p} \\& \quad \leq \int_{0}^{\infty}\ln \biggl[ 1+\frac{\rho}{(U^{\delta }(x)\widetilde{V}_{n})^{\gamma}} \biggr] \biggl[ \frac{U^{\frac{p(1-\delta\sigma)}{q}}(x)f^{p}(x)}{\widetilde{V}_{n}^{1-\sigma}\mu^{\frac{p}{q}}(x)} \biggr] \,dx \\& \quad\quad{} \times \biggl\{ \int_{0}^{\infty}\ln \biggl[ 1+\frac{\rho}{(U^{\delta }(x)\widetilde{V}_{n})^{\gamma}} \biggr] \frac{\widetilde{V}_{n}^{(1-\sigma )(q-1)}\mu(x)}{U^{1-\delta\sigma}(x)}\,dx \biggr\} ^{p-1} \\& \quad =\frac{(\varpi_{\delta}(\sigma,n))^{p-1}}{\widetilde{V}_{n}^{p\sigma -1}\nu_{n}} \int_{0}^{\infty}\ln \biggl[ 1+\frac{\rho}{(U^{\delta}(x)\widetilde{V}_{n})^{\gamma}} \biggr] \frac{U^{(1-\delta\sigma )(p-1)}(x)\nu _{n}}{\widetilde{V}_{n}^{1-\sigma}\mu^{p-1}(x)}f^{p}(x)\,dx. \end{aligned}$$

In view of (15) and Lebesgue term by term integration theorem (*cf.* [32]), we find

24 $$\begin{aligned} J_{1} \leq&\bigl(k(\sigma)\bigr)^{\frac{1}{q}} \Biggl\{ \sum _{n=1}^{\infty } \int_{0}^{\infty}\ln \biggl[ 1+\frac{\rho}{(U^{\delta}(x)\widetilde {V}_{n})^{\gamma}} \biggr] \frac{U^{(1-\delta\sigma)(p-1)}(x)\nu_{n}}{\widetilde{V}_{n}^{1-\sigma}\mu^{p-1}(x)}f^{p}(x)\,dx \Biggr\} ^{\frac{1}{p}} \\ =&\bigl(k(\sigma)\bigr)^{\frac{1}{q}} \Biggl\{ \int_{0}^{\infty}\sum_{n=1}^{\infty } \ln \biggl[ 1+\frac{\rho}{(U^{\delta}(x)\widetilde{V}_{n})^{\gamma}} \biggr] \frac{U^{(1-\delta\sigma)(p-1)}(x)\nu_{n}}{\widetilde{V}_{n}^{1-\sigma}\mu^{p-1}(x)}f^{p}(x)\,dx \Biggr\} ^{\frac{1}{p}} \\ =&\bigl(k(\sigma)\bigr)^{\frac{1}{q}} \biggl[ \int_{0}^{\infty}\omega_{\delta }(\sigma,x) \frac{U^{p(1-\delta\sigma)-1}(x)}{\mu^{p-1}(x)}f^{p}(x)\,dx \biggr] ^{\frac{1}{p}}. \end{aligned}$$

Then by (14), we have (21). By Hölder’s inequality (*cf.* [31]), we have

25 $$\begin{aligned} I =&\sum_{n=1}^{\infty} \biggl\{ \frac{\nu_{n}^{\frac {1}{p}}}{\widetilde{V}_{n}^{\frac{1}{p}-\sigma}} \int_{0}^{\infty}\ln \biggl[ 1+\frac{\rho}{(U^{\delta}(x)\widetilde{V}_{n})^{\gamma}} \biggr] f(x)\,dx \biggr\} \biggl( \frac{\widetilde{V}_{n}^{\frac{1}{p}-\sigma}a_{n}}{\nu_{n}^{\frac {1}{p}}} \biggr) \\ \leq&J_{1} \Vert a \Vert _{q,\widetilde{\Psi}}. \end{aligned}$$

In view of (21), we have (20). On the other hand, assuming that (20) is valid, we set

$$a_{n}:=\frac{\nu_{n}}{\widetilde{V}_{n}^{1-p\sigma}} \biggl\{ \int_{0}^{\infty}\ln \biggl[ 1+\frac{\rho}{(U^{\delta}(x)\widetilde{V} _{n})^{\gamma}} \biggr] f(x)\,dx \biggr\} ^{p-1},\quad n\in\mathbf{N}. $$

Then we find $J_{1}^{p}= \Vert a \Vert _{q,\widetilde{\Psi }}^{q}$. If $J_{1}=0$, then (21) is trivially valid; if $J_{1}=\infty$, then (21) remains impossible. Suppose that $0< J_{1}<\infty$. By (20), we have

$$\Vert a \Vert _{q,\widetilde{\Psi}}^{q}=J_{1}^{p}=I< k( \sigma ) \Vert f \Vert _{p,\Phi_{\delta }} \Vert a \Vert _{q,\widetilde{\Psi}},\quad\quad \Vert a \Vert _{q,\widetilde{\Psi}}^{q-1}=J_{1}< k(\sigma) \Vert f \Vert _{p,\Phi_{\delta}}, $$

and then (21) follows, which is equivalent to (20).

Still by Hölder’s inequality with weight (*cf.* [31]), we have

26 $$\begin{aligned}& \Biggl\{ \sum_{n=1}^{\infty}\ln \biggl[ 1+ \frac{\rho}{(U^{\delta}(x)\widetilde{V}_{n})^{\gamma}} \biggr] a_{n} \Biggr\} ^{q} \\& \quad = \Biggl\{ \sum_{n=1}^{\infty}\ln \biggl[ 1+ \frac{\rho}{(U^{\delta}(x) \widetilde{V}_{n})^{\gamma}} \biggr] \biggl( \frac{U^{\frac{1-\delta \sigma}{q}}(x)\nu_{n}^{\frac{1}{p}}}{\widetilde{V}_{n}^{\frac{1-\sigma}{p}}} \biggr) \biggl( \frac{\widetilde{V}_{n}^{\frac{1-\sigma }{p}}a_{n}}{U^{\frac{1-\delta\sigma}{q}}(x)\nu_{n}^{\frac{1}{p}}} \biggr) \Biggr\} ^{q} \\& \quad \leq \Biggl\{ \sum_{n=1}^{\infty}\ln \biggl[ 1+\frac{\rho}{(U^{\delta }(x)\widetilde{V}_{n})^{\gamma}} \biggr] \frac{U^{(1-\delta\sigma )(p-1)}(x)\nu _{n}}{\widetilde{V}_{n}^{1-\sigma}} \Biggr\} ^{q-1} \\& \quad \quad{} \times\sum_{n=1}^{\infty}\ln \biggl[1+ \frac{\rho}{(U^{\delta}(x)\widetilde{V}_{n})^{\gamma}} \biggr] \frac{\widetilde{V}_{n}^{\frac{q(1-\sigma)}{p}}}{U^{1-\delta\sigma}(x)\nu_{n}^{q-1}}a_{n}^{q} \\& \quad = \frac{(\omega_{\delta}(\sigma,x))^{q-1}}{U^{q\delta\sigma -1}(x)\mu (x)}\sum_{n=1}^{\infty}\ln \biggl[ 1+\frac{\rho}{(U^{\delta }(x)\widetilde{V}_{n})^{\gamma}} \biggr] \frac{\widetilde{V}_{n}^{(1-\sigma)(q-1)}\mu (x)}{U^{1-\delta\sigma}(x)\nu_{n}^{q-1}}a_{n}^{q}. \end{aligned}$$

Then by (14) and Lebesgue term by term integration theorem (*cf.* [32]), it follows that

27 $$\begin{aligned} J_{2} < &\bigl(k(\sigma)\bigr)^{\frac{1}{p}} \Biggl\{ \int_{0}^{\infty}\sum_{n=1}^{\infty } \ln \biggl[ 1+\frac{\rho}{(U^{\delta}(x)\widetilde{V}_{n})^{\gamma}} \biggr] \frac{\widetilde{V}_{n}^{(1-\sigma)(q-1)}\mu(x)}{U^{1-\delta \sigma}(x)\nu_{n}^{q-1}}a_{n}^{q}\,dx \Biggr\} ^{\frac{1}{q}} \\ =&\bigl(k(\sigma)\bigr)^{\frac{1}{p}} \Biggl\{ \sum _{n=1}^{\infty} \int _{0}^{\infty }\ln \biggl[1+\frac{\rho}{(U^{\delta}(x)\widetilde{V}_{n})^{\gamma }} \biggr] \frac{\widetilde{V}_{n}^{(1-\sigma)(q-1)}\mu(x)}{U^{1-\delta\sigma }(x)\nu_{n}^{q-1}}a_{n}^{q}\,dx \Biggr\} ^{\frac{1}{q}} \\ =&\bigl(k(\sigma)\bigr)^{\frac{1}{p}} \Biggl[ \sum _{n=1}^{\infty}\varpi_{\delta }(\sigma,n) \frac{\widetilde{V}_{n}^{q(1-\sigma)-1}}{\nu_{n}^{q-1}}a_{n}^{q} \Biggr] ^{\frac{1}{q}}. \end{aligned}$$

In view of (15), we have (22). By Hölder’s inequality (*cf.* [31]), we have

28 $$\begin{aligned} I =& \int_{0}^{\infty} \biggl( \frac{U^{\frac{1}{q}-\delta\sigma }(x)}{\mu^{\frac{1}{q}}(x)}f(x) \biggr) \Biggl\{ \frac{\mu^{\frac {1}{q}}(x)}{U^{\frac{1}{q}-\delta\sigma}(x)}\sum_{n=1}^{\infty} \ln \biggl[ 1+\frac{\rho}{(U^{\delta}(x)\widetilde{V}_{n})^{\gamma}} \biggr] a_{n} \Biggr\} \,dx \\ \leq& \Vert f \Vert _{p,\Phi_{\delta}}J_{2}. \end{aligned}$$

Then by (22), we have (20). On the other hand, assuming that (22) is valid, we set

$$f(x):=\frac{\mu(x)}{U^{1-q\delta\sigma}(x)} \Biggl\{ \sum_{n=1}^{\infty } \ln \biggl[1+\frac{\rho}{(U^{\delta}(x)\widetilde{V}_{n})^{\gamma }} \biggr] a_{n} \Biggr\} ^{q-1},\quad x\in\mathbf{R}_{+}. $$

Then we find $J_{2}^{q}= \Vert f \Vert _{p,\Phi_{\delta }}^{p}$. If $J_{2}=0$, then (22) is trivially valid; if $J_{2}=\infty$, then (22) keeps impossible. Suppose that $0< J_{2}<\infty$. By (20), we have

$$\Vert f \Vert _{p,\Phi_{\delta}}^{p}=J_{2}^{q}=I< k( \sigma ) \Vert f \Vert _{p,\Phi_{\delta }} \Vert a \Vert _{q,\widetilde{\Psi}},\quad\quad \Vert f \Vert _{p,\Phi_{\delta}}^{p-1}=J_{2}< k(\sigma ) \Vert a \Vert _{q,\widetilde{\Psi}}, $$

and then (22) follows, which is equivalent to (20).

Therefore, inequalities (20), (21) and (22) are equivalent. The theorem is proved. □

### Theorem 2

*As regards the assumptions of Theorem* 1, *if there exists a* $n_{0}\in\mathbf{N}$, *such that* $\nu_{n}\geq\nu_{n+1}$ ($n\in \{n_{0},n_{0}+1,\ldots\}$), *and* $U(\infty)=V_{\infty}=\infty$, *then the constant factor* $k(\sigma)$ *in* (20), (21) *and* (22) *is the best possible*.

### Proof

For $\varepsilon\in(0,q\sigma)$, we set $\widetilde {\sigma }=\sigma-\frac{\varepsilon}{q}$ ($<\min\{1,\gamma\}$), and $\widetilde{f}=\widetilde{f}(x)$, $x\in\mathbf{R}_{+}$, $\widetilde{a}=\{\widetilde{a}_{n}\}_{n=1}^{\infty}$,

29 $$\begin{aligned}& \widetilde{f}(x) = \textstyle\begin{cases} U^{\delta(\widetilde{\sigma}+\varepsilon)-1}(x)\mu(x),& 0< x^{\delta }\leq1, \\ 0,& x^{\delta}>1,\end{cases}\displaystyle \end{aligned}$$

30 $$\begin{aligned}& \widetilde{a}_{n} =\widetilde{V}_{n}^{\widetilde{\sigma}-1} \nu_{n}=\widetilde{V}_{n}^{\sigma-\frac{\varepsilon}{q}-1} \nu_{n},\quad n\in \mathbf{N}. \end{aligned}$$

Then, for $\delta=\pm1$, since $U(\infty)=\infty$, we obtain

31 $$ \int_{\{x>0;0< x^{\delta}\leq1\}}\frac{\mu(x)}{U^{1-\delta \varepsilon}(x)}\,dx=\frac{1}{\varepsilon}U^{\delta\varepsilon}(1). $$

By (31), (19) and (17), we find

32 $$\begin{aligned}& \begin{aligned}[b] \Vert \widetilde{f} \Vert _{p,\Phi_{\delta}} \Vert \widetilde{a} \Vert _{q,\widetilde{\Psi}} &= \biggl( \int_{\{x>0;0< x^{\delta}\leq1\}}\frac{\mu(x)\,dx}{U^{1-\delta \varepsilon}(x)} \biggr) ^{\frac{1}{p}} \Biggl( \sum_{n=1}^{\infty}\frac {\nu _{n}}{\widetilde{V}_{n}^{1+\varepsilon}} \Biggr) ^{\frac{1}{q}} \\ &=\frac{1}{\varepsilon}U^{\frac{\delta\varepsilon}{p}}(1) \biggl( \frac{1}{\widetilde{V}_{n_{0}}^{\varepsilon}}+ \varepsilon O(1) \biggr) ^{\frac{1}{q}}, \end{aligned} \\& \widetilde{I} := \int_{0}^{\infty}\sum_{n=1}^{\infty} \ln \biggl[ 1+\frac{\rho}{(U^{\delta}(x)\widetilde{V}_{n})^{\gamma}} \biggr] \widetilde {a}_{n}\widetilde{f}(x)\,dx \\& \hphantom{\widetilde{I}}= \int_{\{x>0;0< x^{\delta}\leq1\}}\sum_{n=1}^{\infty} \ln \biggl[ 1+\frac{\rho}{(U^{\delta}(x)\widetilde{V}_{n})^{\gamma}} \biggr] \frac {\widetilde{V}_{n}^{\widetilde{\sigma}-1}\nu_{n}\mu(x)}{U^{1-\delta(\widetilde {\sigma }+\varepsilon)}(x)}\,dx \\& \hphantom{\widetilde{I}}= \int_{\{x>0;0< x^{\delta}\leq1\}}\omega_{\delta}(\widetilde { \sigma},x)\frac{\mu(x)}{U^{1-\delta\varepsilon}(x)}\,dx \\& \hphantom{\widetilde{I}} \geq k(\widetilde{\sigma}) \int_{\{x>0;0< x^{\delta}\leq1\}}\bigl(1-\theta _{\delta}(\widetilde{\sigma},x) \bigr)\frac{\mu(x)}{U^{1-\delta \varepsilon}(x)}\,dx \\& \hphantom{\widetilde{I}}= k(\widetilde{\sigma}) \int_{\{x>0;0< x^{\delta}\leq 1\}}\bigl(1-O\bigl(\bigl(U(x)\bigr)^{\delta(\frac{\sigma}{2}-\frac{\varepsilon }{2q})}\bigr) \bigr)\frac{\mu(x)}{U^{1-\delta\varepsilon}(x)}\,dx \\& \hphantom{\widetilde{I}} = k(\widetilde{\sigma}) \biggl[ \int_{\{x>0;0< x^{\delta}\leq1\}}\frac {\mu (x)}{U^{1-\delta\varepsilon}(x)}\,dx \\& \hphantom{\widetilde{I}}\quad{} - \int_{\{x>0;0< x^{\delta}\leq1\}}O\biggl(\frac{\mu (x)}{U^{1-\delta(\frac{\sigma}{2}+\frac{\varepsilon}{p}+\frac{\varepsilon }{2q})}(x)}\biggr)\,dx \biggr] \\& \hphantom{\widetilde{I}} = \frac{1}{\varepsilon}k\biggl(\sigma-\frac{\varepsilon}{q}\biggr) \bigl(U^{\delta \varepsilon}(1)-\varepsilon O_{1}(1)\bigr). \end{aligned}$$

If there exists a positive constant $K\leq k(\sigma)$, such that (20) is valid when replacing $k(\sigma)$ to *K*, then in particular, by Lebesgue term by term integration theorem, we have $\varepsilon \widetilde{I}<\varepsilon K \Vert \widetilde{f} \Vert _{p,\Phi_{\delta }} \Vert \widetilde{a} \Vert _{q,\widetilde{\Psi}}$, namely,

$$k\biggl(\sigma-\frac{\varepsilon}{q}\biggr) \bigl(U^{\delta\varepsilon}(1)-\varepsilon O_{1}(1)\bigr)< K\cdot U^{\frac{\delta\varepsilon}{p}}(1) \biggl( \frac{1}{\widetilde{V}_{n_{0}}^{\varepsilon}}+\varepsilon O(1) \biggr) ^{\frac{1}{q}}. $$

It follows that $k(\sigma)\leq K$ ($\varepsilon\rightarrow0^{+}$). Hence, $K=k(\sigma)$ is the best possible constant factor of (20).

The constant factor $k(\sigma)$ in (21) ((22)) is still the best possible. Otherwise, we would reach a contradiction by (25) ((28)) that the constant factor in (20) is not the best possible. The theorem is proved. □

For $p>1$, we find $\widetilde{\Psi}^{1-p}(n)=\frac{\nu _{n}}{\widetilde{V}_{n}^{1-p\sigma}}$ ($n\in\mathbf{N}$), $\Phi_{\delta}^{1-q}(x)=\frac {\mu (x)}{U^{1-q\delta\sigma}(x)}$ ($x\in\mathbf{R}_{+}$), and define the following real normed spaces:

$$\begin{aligned}& L_{p,\Phi_{\delta}}(\mathbf{R}_{+}) = \bigl\{ f;f=f(x),x\in \mathbf{R}_{+}, \Vert f \Vert _{p,\Phi_{\delta}}< \infty \bigr\} , \\& l_{q,\widetilde{\Psi}} = \bigl\{ a;a=\{a_{n}\}_{n=1}^{\infty}, \Vert a \Vert _{q,\widetilde{\Psi}}< \infty\bigr\} , \\& L_{q,\Phi_{\delta}^{1-q}}(\mathbf{R}_{+}) = \bigl\{ h;h=h(x),x\in\mathbf {R}_{+}, \Vert h \Vert _{q,\Phi_{\delta}^{1-q}}< \infty\bigr\} , \\& l_{p,\widetilde{\Psi}^{1-p}} = \bigl\{ c;c=\{c_{n}\}_{n=1}^{\infty}, \Vert c \Vert _{p,\widetilde{\Psi}^{1-p}}< \infty\bigr\} . \end{aligned}$$

Assuming that $f\in L_{p,\Phi_{\delta}}(\mathbf{R}_{+})$, setting

$$c=\{c_{n}\}_{n=1}^{\infty},\quad\quad c_{n}:= \int_{0}^{\infty}\ln \biggl[ 1+\frac {\rho }{(U^{\delta}(x)\widetilde{V}_{n})^{\gamma}} \biggr] f(x)\,dx,\quad n\in \mathbf{N}, $$

we can rewrite (21) as $\Vert c \Vert _{p,\widetilde {\Psi}^{1-p}}< k(\sigma ) \Vert f \Vert _{p,\Phi_{\delta}}<\infty$, namely, $c\in l_{p,\widetilde{\Psi}^{1-p}}$.

### Definition 1

Define a half-discrete Hardy-Hilbert-type operator $T_{1}:L_{p,\Phi_{\delta}}(\mathbf{R}_{+})\rightarrow l_{p,\widetilde {\Psi}^{1-p}}$ as follows: For any $f\in L_{p,\Phi_{\delta}}(\mathbf{R}_{+})$, there exists a unique representation $T_{1}f=c\in l_{p,\widetilde{\Psi} ^{1-p}}$. Define the formal inner product of $T_{1}f$ and $a=\{a_{n}\}_{n=1}^{\infty}\in l_{q,\widetilde{\Psi}}$ as follows:

33 $$ (T_{1}f,a):=\sum_{n=1}^{\infty} \biggl\{ \int_{0}^{\infty}\ln \biggl[ 1+\frac{\rho}{(U^{\delta}(x)\widetilde{V}_{n})^{\gamma}} \biggr] f(x)\,dx \biggr\} a_{n}. $$

Then we can rewrite (20) and (21) as follows:

34 $$\begin{aligned}& (T_{1}f,a) < k(\sigma) \Vert f \Vert _{p,\Phi_{\delta }} \Vert a \Vert _{q,\widetilde{\Psi}}, \end{aligned}$$

35 $$\begin{aligned}& \Vert T_{1}f \Vert _{p,\widetilde{\Psi}^{1-p}} < k(\sigma ) \Vert f \Vert _{p,\Phi_{\delta }}. \end{aligned}$$

Define the norm of operator $T_{1}$ as follows:

$$\Vert T_{1} \Vert :=\sup_{f(\neq\theta)\in L_{p,\Phi _{\delta}}(\mathbf{R}_{+})}\frac{ \Vert T_{1}f \Vert _{p,\widetilde{\Psi}^{1-p}}}{ \Vert f \Vert _{p,\Phi_{\delta}}}. $$

Then by (35), it follows that $\Vert T_{1} \Vert \leq k(\sigma)$. Since by Theorem 2, the constant factor in (35) is the best possible, we have

36 $$ \Vert T_{1} \Vert =k(\sigma)=\frac{\rho^{\sigma/\gamma }\pi}{\sigma\sin(\frac{\pi\sigma}{\gamma})}. $$

Assuming that $a=\{a_{n}\}_{n=1}^{\infty}\in l_{q,\widetilde{\Psi}}$, setting

$$h(x):=\sum_{n=1}^{\infty}\ln \biggl[ 1+ \frac{\rho}{(U^{\delta}(x)\widetilde{V}_{n})^{\gamma}} \biggr] a_{n},\quad x\in\mathbf{R}_{+}, $$

we can rewrite (22) as $\Vert h \Vert _{q,\Phi _{\delta}^{1-q}}< k(\sigma ) \Vert a \Vert _{q,\widetilde{\Psi}}<\infty$, namely, $h\in L_{q,\Phi_{\delta }^{1-q}}(\mathbf{R}_{+})$.

### Definition 2

Define a half-discrete Hardy-Hilbert-type operator $T_{2}:l_{q,\widetilde{\Psi}}\rightarrow L_{q,\Phi_{\delta }^{1-q}}(\mathbf{R}_{+})$ as follows: For any $a=\{a_{n}\}_{n=1}^{\infty}\in l_{q,\widetilde{\Psi}}$, there exists a unique representation $T_{2}a=h\in L_{q,\Phi _{\delta}^{1-q}}(\mathbf{R}_{+})$. Define the formal inner product of $T_{2}a$ and $f\in L_{p,\Phi_{\delta}}(\mathbf{R}_{+})$ as follows:

37 $$ (T_{2}a,f):= \int_{0}^{\infty} \Biggl\{ \sum _{n=1}^{\infty}\ln \biggl[ 1+\frac{\rho}{(U^{\delta}(x)\widetilde{V}_{n})^{\gamma}} \biggr] a_{n} \Biggr\} f(x)\,dx. $$

Then we can rewrite (20) and (22) as follows:

38 $$\begin{aligned}& (T_{2}a,f) < k(\sigma) \Vert f \Vert _{p,\Phi_{\delta }} \Vert a \Vert _{q,\widetilde{\Psi}}, \end{aligned}$$

39 $$\begin{aligned}& \Vert T_{2}a \Vert _{q,\Phi_{\delta}^{1-q}} < k(\sigma ) \Vert a \Vert _{q,\widetilde{\Psi}}. \end{aligned}$$

Define the norm of operator $T_{2}$ as follows:

$$\Vert T_{2} \Vert :=\sup_{a(\neq\theta)\in l_{q,\widetilde {\Psi}}} \frac{ \Vert T_{2}a \Vert _{q,\Phi_{\delta}^{1-q}}}{ \Vert a \Vert _{q,\widetilde{\Psi}}}. $$

Then by (39), we find $\Vert T_{2} \Vert \leq k(\sigma)$. Since, by Theorem 2, the constant factor in (39) is the best possible, we have

40 $$ \Vert T_{2} \Vert =k(\sigma)=\frac{\rho^{\sigma/\gamma }\pi}{\sigma\sin(\frac{\pi\sigma}{\gamma})}= \Vert T_{1} \Vert . $$

## Some equivalent reverses

In the following, we also set

$$\begin{aligned}& \widetilde{\Phi}_{\delta}(x) : =\bigl(1-\theta_{\delta}(\sigma,x) \bigr)\frac {U^{p(1-\delta\sigma)-1}(x)}{\mu^{p-1}(x)} \quad (x\in\mathbf{R}_{+}), \\& \Psi(n) : =\frac{V_{n}^{q(1-\sigma)-1}}{\nu_{n}^{q-1}} \quad (n\in \mathbf{N}). \end{aligned}$$

For $0< p<1$ or $p<0$, we still use the formal symbols $\Vert f \Vert _{p,\Phi _{\delta}}$, $\Vert f \Vert _{p,\widetilde{\Phi}_{\delta }}$ and $\Vert a \Vert _{q,\widetilde{\Psi}}$.

### Theorem 3

*If* $\rho>0$, $k(\sigma)$ *is determined by* (9), *there exists a* $n_{0}\in\mathbf{N}$, *such that* $\nu_{n}\geq\nu_{n+1}$ ($n\in\{n_{0},n_{0}+1,\ldots \}$), *and* $U(\infty)=V_{\infty}=\infty$, *then*, *for* $p<0$, $0< \Vert f \Vert _{p,\Phi _{\delta}}, \Vert a \Vert _{q,\widetilde{\Psi}}<\infty$, *we have the following equivalent inequalities with the best possible constant factor* $k(\sigma)$:

41 $$\begin{aligned}& I=\sum_{n=1}^{\infty} \int_{0}^{\infty}\ln \biggl[ 1+\frac{\rho }{(U^{\delta }(x)\widetilde{V}_{n})^{\gamma}} \biggr] a_{n}f(x)\,dx>k(\sigma) \Vert f \Vert _{p,\Phi _{\delta}} \Vert a \Vert _{q,\widetilde{\Psi}}, \end{aligned}$$

42 $$\begin{aligned}& \begin{aligned}[b] J_{1} &= \Biggl\{ \sum_{n=1}^{\infty} \frac{\nu_{n}}{\widetilde{V}_{n}^{1-p\sigma}} \biggl[ \int_{0}^{\infty}\ln \biggl( 1+\frac{\rho}{(U^{\delta}(x)\widetilde{V}_{n})^{\gamma}} \biggr) f(x)\,dx \biggr] ^{p} \Biggr\} ^{\frac{1}{p}} \\ &>k(\sigma) \Vert f \Vert _{p,\Phi_{\delta}}, \end{aligned} \end{aligned}$$

43 $$\begin{aligned}& \begin{aligned}[b] J_{2} &= \Biggl\{ \int_{0}^{\infty}\frac{\mu(x)}{U^{1-q\delta\sigma }(x)} \Biggl[ \sum_{n=1}^{\infty}\ln \biggl( 1+ \frac{\rho}{(U^{\delta}(x)\widetilde{V}_{n})^{\gamma}} \biggr) a_{n} \Biggr] ^{q}\,dx \Biggr\} ^{\frac {1}{q}} \\ &>k(\sigma) \Vert a \Vert _{q,\widetilde{\Psi}}. \end{aligned} \end{aligned}$$

### Proof

By the reverse Hölder inequality with weight (*cf.* [31]), since $p<0$, in a similar way to obtaining (23) and (24), we have

$$\begin{aligned}& \biggl\{ \int_{0}^{\infty}\ln \biggl[1+\frac{\rho}{(U^{\delta}(x)\widetilde{V}_{n})^{\gamma}} \biggr] f(x)\,dx \biggr\} ^{p} \\& \quad \leq \frac{(\varpi_{\delta}(\sigma,n))^{p-1}}{\widetilde{V}_{n}^{p\sigma-1}\nu_{n}} \int_{0}^{\infty}\ln \biggl[ 1+\frac{\rho}{(U^{\delta}(x)\widetilde{V}_{n})^{\gamma}} \biggr]\frac{U^{(1-\delta \sigma )(p-1)}(x)\nu_{n}}{\widetilde{V}_{n}^{1-\sigma}\mu^{p-1}(x)}f^{p}(x)\,dx. \end{aligned}$$

Then by the note of Lemma 2 and the Lebesgue term by term integration theorem, it follows that

$$\begin{aligned} J_{1} \geq&\bigl(k(\sigma)\bigr)^{\frac{1}{q}} \Biggl\{ \sum _{n=1}^{\infty } \int_{0}^{\infty}\ln \biggl[ 1+\frac{\rho}{(U^{\delta}(x)\widetilde {V}_{n})^{\gamma}} \biggr] \frac{U^{(1-\delta\sigma)(p-1)}(x)\nu_{n}}{\widetilde{V}_{n}^{1-\sigma}\mu^{p-1}(x)}f^{p}(x)\,dx \Biggr\} ^{\frac{1}{p}} \\ =&\bigl(k(\sigma)\bigr)^{\frac{1}{q}} \biggl[ \int_{0}^{\infty}\omega_{\delta }(\sigma,x) \frac{U^{p(1-\delta\sigma)-1}(x)}{\mu^{p-1}(x)}f^{p}(x)\,dx \biggr] ^{\frac{1}{p}}. \end{aligned}$$

In view of (14), we have (42). By the reverse Hölder inequality (*cf.* [31]), we have

44 $$\begin{aligned} I =&\sum_{n=1}^{\infty} \biggl\{ \frac{\nu_{n}^{\frac {1}{p}}}{\widetilde{V}_{n}^{\frac{1}{p}-\sigma}} \int_{0}^{\infty}\ln \biggl[ 1+\frac{\rho}{(U^{\delta}(x)\widetilde{V}_{n})^{\gamma}} \biggr] f(x)\,dx \biggr\} \biggl( \frac{\widetilde{V}_{n}^{\frac{1}{p}-\sigma}a_{n}}{\nu_{n}^{\frac {1}{p}}} \biggr) \\ \geq&J_{1} \Vert a \Vert _{q,\widetilde{\Psi}}. \end{aligned}$$

Then by (42), we have (41). On the other hand, assuming that (41) is valid, we set $a_{n}$ as in Theorem 1. Then we find $J_{1}^{p}= \Vert a \Vert _{q,\widetilde{\Psi}}^{q}$. If $J_{1}=\infty$, then (42) is trivially valid; if $J_{1}=0$, then (42) remains impossible. Suppose that $0< J_{1}<\infty$. By (41), it follows that

$$\Vert a \Vert _{q,\widetilde{\Psi}}^{q}=J_{1}^{p}=I>k( \sigma ) \Vert f \Vert _{p,\Phi_{\delta }} \Vert a \Vert _{q,\widetilde{\Psi}},\quad\quad \Vert a \Vert _{q,\widetilde{\Psi}}^{q-1}=J_{1}>k(\sigma) \Vert f \Vert _{p,\Phi_{\delta}}, $$

and then (42) follows, which is equivalent to (41).

Still by the reverse Hölder inequality with weight (*cf.* [31]), since $0< q<1$, in a similar way to obtaining (26) and (27), we have

$$\begin{aligned}& \Biggl\{ \sum_{n=1}^{\infty}\ln \biggl[ 1+ \frac{\rho}{(U^{\delta}(x)\widetilde{V}_{n})^{\gamma}} \biggr] a_{n} \Biggr\} ^{q} \\& \quad \geq \frac{(\omega_{\delta}(\sigma,x))^{q-1}}{U^{q\delta\sigma -1}(x)\mu(x)}\sum_{n=1}^{\infty}\ln \biggl[ 1+\frac{\rho}{(U^{\delta }(x)\widetilde{V}_{n})^{\gamma}} \biggr] \frac{\widetilde{V}_{n}^{(1-\sigma )(q-1)}\mu(x)}{U^{1-\delta\sigma}(x)\nu_{n}^{q-1}}a_{n}^{q}. \end{aligned}$$

Then by (14) and the Lebesgue term by term integration theorem (*cf.* [32]), it follows that

$$\begin{aligned} J_{2} >&\bigl(k(\sigma)\bigr)^{\frac{1}{p}} \Biggl\{ \int_{0}^{\infty }\sum_{n=1}^{\infty} \ln \biggl[ 1+\frac{\rho}{(U^{\delta}(x)\widetilde {V}_{n})^{\gamma}} \biggr] \frac{\widetilde{V}_{n}^{(1-\sigma)(q-1)}\mu (x)}{U^{1-\delta\sigma}(x)\nu_{n}^{q-1}}a_{n}^{q}\,dx \Biggr\} ^{\frac {1}{q}} \\ =&\bigl(k(\sigma)\bigr)^{\frac{1}{p}} \Biggl[ \sum _{n=1}^{\infty}\varpi_{\delta }(\sigma,n) \frac{\widetilde{V}_{n}^{q(1-\sigma)-1}}{\nu_{n}^{q-1}}a_{n}^{q} \Biggr] ^{\frac{1}{q}}. \end{aligned}$$

In view of the note of Lemma 2, we have (43). By the reverse Hölder inequality, we have

45 $$\begin{aligned} I =& \int_{0}^{\infty}\frac{U^{\frac{1}{q}-\delta\sigma}(x)}{\mu ^{\frac{1}{q}}(x)}f(x) \Biggl\{ \frac{\mu^{\frac{1}{q}}(x)}{U^{\frac{1}{q}-\delta \sigma}(x)}\sum_{n=1}^{\infty}\ln \biggl[ 1+\frac{\rho}{(U^{\delta }(x)\widetilde{V}_{n})^{\gamma}} \biggr] a_{n} \Biggr\} \,dx \\ \geq& \Vert f \Vert _{p,\Phi_{\delta}}J_{2}. \end{aligned}$$

Then by (43), we have (41). On the other hand, assuming that (43) is valid, we set $f(x)$ as in Theorem 1. Then we find $J_{2}^{q}= \Vert f \Vert _{p,\Phi_{\delta}}^{p}$. If $J_{2}=\infty$, then (43) is trivially valid; if $J_{2}=0$, then (43) remains impossible. Suppose that $0< J_{2}<\infty$. By (41), it follows that

$$\Vert f \Vert _{p,\Phi_{\delta}}^{p}=J_{2}^{q}=I>k( \sigma ) \Vert f \Vert _{p,\Phi_{\delta }} \Vert a \Vert _{q,\widetilde{\Psi}},\quad\quad \Vert f \Vert _{p,\Phi_{\delta}}^{p-1}=J_{2}>k(\sigma ) \Vert a \Vert _{q,\widetilde{\Psi}}, $$

and then (43) follows, which is equivalent to (41).

Therefore, inequalities (41), (42) and (43) are equivalent.

For $\varepsilon\in(0,q\sigma)$, we set $\widetilde{\sigma}=\sigma- \frac{\varepsilon}{q}$, and $\widetilde{f}=\widetilde{f}(x)$, $x\in \mathbf{R}_{+}$, $\widetilde{a}=\{\widetilde{a}_{n}\}_{n=1}^{\infty}$,

$$\begin{aligned}& \widetilde{f}(x) = \textstyle\begin{cases} U^{\delta(\widetilde{\sigma}+\varepsilon)-1}(x)\mu(x),&0< x^{\delta }\leq1, \\ 0,&x^{\delta}>0,\end{cases}\displaystyle \\& \widetilde{a}_{n} =\widetilde{V}_{n}^{\widetilde{\sigma}-1} \nu_{n}=\widetilde{V}_{n}^{\sigma-\frac{\varepsilon}{q}-1} \nu_{n},\quad n\in \mathbf{N}. \end{aligned}$$

By (19), (31) and (14), we obtain

$$\begin{aligned}& \Vert \widetilde{f} \Vert _{p,\Phi_{\delta}} \Vert \widetilde{a} \Vert _{q,\widetilde{\Psi}}=\frac{1}{\varepsilon}U^{\frac{\delta\varepsilon}{p}}(1) \biggl( \frac {1}{\widetilde{V}_{n_{0}}^{\varepsilon}}+\varepsilon O(1) \biggr) ^{\frac{1}{q}}, \\& \begin{aligned} \widetilde{I} &=\sum_{n=1}^{\infty} \int_{0}^{\infty}\ln \biggl[ 1+\frac{\rho}{(U^{\delta}(x)\widetilde{V}_{n})^{\gamma}} \biggr] \widetilde {a}_{n}\widetilde{f}(x)\,dx \\ &= \int_{\{x>0;0< x^{\delta}\leq1\}}\omega_{\delta}(\widetilde { \sigma},x)\frac{\mu(x)}{U^{1-\delta\varepsilon}(x)}\,dx \\ &\leq k(\widetilde{\sigma}) \int_{\{x>0;0< x^{\delta}\leq1\}}\frac {\mu(x)}{U^{1-\delta\varepsilon}(x)}\,dx=\frac{1}{\varepsilon}k\biggl( \sigma-\frac{ \varepsilon}{q}\biggr)U^{\delta\varepsilon}(1). \end{aligned} \end{aligned}$$

If there exists a positive constant $K\geq k(\sigma)$, such that (41) is valid when replacing $k(\sigma)$ to *K*, then in particular, we have $\varepsilon\widetilde{I}>\varepsilon K \Vert \widetilde{f} \Vert _{p,\Phi_{\delta }} \Vert \widetilde{a} \Vert _{q,\widetilde{\Psi}}$, namely,

$$k\biggl(\sigma-\frac{\varepsilon}{q}\biggr)U^{\delta\varepsilon}(1)>K\cdot U^{\frac{\delta\varepsilon}{p}}(1) \biggl( \frac{1}{\widetilde{V}_{n_{0}}^{\varepsilon}}+\varepsilon O(1) \biggr) ^{\frac{1}{q}}. $$

It follows that $k(\sigma)\geq K$ ($\varepsilon\rightarrow0^{+}$). Hence, $K=k(\sigma)$ is the best possible constant factor of (41). The constant factor $k(\sigma)$ in (42) ((43)) is still the best possible. Otherwise, we would reach the contradiction by (44) ((45)) that the constant factor in (41) is not the best possible. The theorem is proved. □

### Theorem 4

*As regards the assumptions of Theorem* 3, *if* $0< p<1$, $0< \Vert f \Vert _{p,\Phi_{\delta}}$, $\Vert a \Vert _{q,\widetilde{\Psi}}<\infty$, *then we have the following equivalent inequalities with the best possible constant factor* $k(\sigma)$:

46 $$\begin{aligned}& I=\sum_{n=1}^{\infty} \int_{0}^{\infty}\ln \biggl[ 1+\frac{\rho }{(U^{\delta }(x)\widetilde{V}_{n})^{\gamma}} \biggr] a_{n}f(x)\,dx>k(\sigma) \Vert f \Vert _{p,\widetilde{\Phi}_{\delta}} \Vert a \Vert _{q,\widetilde{\Psi }}, \end{aligned}$$

47 $$\begin{aligned}& \begin{aligned}[b] J_{1} &= \Biggl\{ \sum_{n=1}^{\infty} \frac{\nu_{n}}{\widetilde{V}_{n}^{1-p\sigma}} \biggl[ \int_{0}^{\infty}\ln \biggl( 1+\frac{\rho}{(U^{\delta}(x)\widetilde{V}_{n})^{\gamma}} \biggr) f(x)\,dx \biggr] ^{p} \Biggr\} ^{\frac{1}{p}} \\ &>k(\sigma) \Vert f \Vert _{p,\widetilde{\Phi}_{\delta}}, \end{aligned} \end{aligned}$$

48 $$\begin{aligned}& \begin{aligned}[b] J &:= \Biggl\{ \int_{0}^{\infty}\frac{U^{q\delta\sigma-1}(x)\mu(x)}{(1-\theta_{\delta}(\sigma,x))^{q-1}} \Biggl[ \sum _{n=1}^{\infty}\ln \biggl( 1+\frac{\rho}{(U^{\delta}(x)\widetilde{V}_{n})^{\gamma}} \biggr) a_{n} \Biggr] ^{q}\,dx \Biggr\} ^{\frac{1}{q}} \\ &>k(\sigma) \Vert a \Vert _{q,\widetilde{\Psi}}. \end{aligned} \end{aligned}$$

### Proof

By the reverse Hölder inequality with weight (*cf.* [31]), since $0< p<1$, in a similar way to obtaining (23) and (24), we have

$$\begin{aligned}& \biggl\{ \int_{0}^{\infty}\ln \biggl[ 1+\frac{\rho}{(U^{\delta}(x)\widetilde{V}_{n})^{\gamma}} \biggr] f(x)\,dx \biggr\} ^{p} \\ & \quad \geq \frac{(\varpi_{\delta}(\sigma,n))^{p-1}}{\widetilde{V}_{n}^{p\sigma-1}\nu_{n}} \int_{0}^{\infty}\ln \biggl[ 1+\frac{\rho}{(U^{\delta}(x)\widetilde{V}_{n})^{\gamma}} \biggr] \frac{U^{(1-\delta \sigma)(p-1)}(x)\nu_{n}}{\widetilde{V}_{n}^{1-\sigma}\mu^{p-1}(x)}f^{p}(x)\,dx. \end{aligned}$$

In view of the note of Lemma 2 and the Lebesgue term by term integration theorem (*cf.* [32]), we find

$$\begin{aligned} J_{1} \geq&\bigl(k(\sigma)\bigr)^{\frac{1}{q}} \Biggl\{ \sum _{n=1}^{\infty } \int_{0}^{\infty}\ln \biggl[ 1+\frac{\rho}{(U^{\delta}(x)\widetilde {V}_{n})^{\gamma}} \biggr] \frac{U^{(1-\delta\sigma)(p-1)}(x)\nu_{n}}{\widetilde{V}_{n}^{1-\sigma}\mu^{p-1}(x)}f^{p}(x)\,dx \Biggr\} ^{\frac{1}{p}} \\ =&\bigl(k(\sigma)\bigr)^{\frac{1}{q}} \biggl[ \int_{0}^{\infty}\omega_{\delta }(\sigma,x) \frac{U^{p(1-\delta\sigma)-1}(x)}{\mu^{p-1}(x)}f^{p}(x)\,dx \biggr] ^{\frac{1}{p}}. \end{aligned}$$

Then by (17), we have (47). By the reverse Hölder inequality, we have

49 $$\begin{aligned} I =&\sum_{n=1}^{\infty} \biggl\{ \frac{\nu_{n}^{\frac {1}{p}}}{\widetilde{V}_{n}^{\frac{1}{p}-\sigma}} \int_{0}^{\infty}\ln \biggl[ 1+\frac{\rho}{(U^{\delta}(x)\widetilde{V}_{n})^{\gamma}} \biggr] f(x)\,dx \biggr\} \biggl( \frac{\widetilde{V}_{n}^{ \frac{1}{p}-\sigma}a_{n}}{\nu_{n}^{\frac {1}{p}}} \biggr) \\ \geq&J_{1} \Vert a \Vert _{q,\widetilde{\Psi}}. \end{aligned}$$

Then by (47), we have (46). On the other hand, assuming that (46) is valid, we set $a_{n}$ as in Theorem 1. Then we find $J_{1}^{p}= \Vert a \Vert _{q,\widetilde{\Psi}}^{q}$. If $J_{1}=\infty$, then (47) is trivially valid; if $J_{1}=0$, then (47) remains impossible. Suppose that $0< J_{1}<\infty$. By (46), it follows that

$$\Vert a \Vert _{q,\widetilde{\Psi}}^{q}=J_{1}^{p}=I>k( \sigma ) \Vert f \Vert _{p,\widetilde{\Phi}_{\delta}} \Vert a \Vert _{q,\widetilde{\Psi}},\quad\quad \Vert a \Vert _{q,\widetilde{\Psi}}^{q-1}=J_{1}>k(\sigma) \Vert f \Vert _{p,\widetilde{\Phi }_{\delta}}, $$

and then (47) follows, which is equivalent to (46).

Still by the reverse Hölder inequality with weight (*cf.* [31]), since $q<0$, we have

$$\begin{aligned}& \Biggl\{ \sum_{n=1}^{\infty}\ln \biggl[ 1+ \frac{\rho}{(U^{\delta}(x)\widetilde{V}_{n})^{\gamma}} \biggr] a_{n} \Biggr\} ^{q} \\& \quad \leq \frac{(\omega_{\delta}(\sigma,x))^{q-1}}{U^{q\delta\sigma -1}(x)\mu(x)}\sum_{n=1}^{\infty}\ln \biggl[ 1+\frac{\rho}{(U^{\delta }(x)\widetilde{V}_{n})^{\gamma}} \biggr] \frac{\widetilde{V}_{n}^{(1-\sigma )(q-1)}\mu(x)}{U^{1-\delta\sigma}(x)\nu_{n}^{q-1}}a_{n}^{q}. \end{aligned}$$

Then by (17) and the Lebesgue term by term integration theorem, it follows that

$$\begin{aligned} J >&\bigl(k(\sigma)\bigr)^{\frac{1}{p}} \Biggl\{ \int_{0}^{\infty}\sum_{n=1}^{\infty } \ln \biggl[1+\frac{\rho}{(U^{\delta}(x)\widetilde{V}_{n})^{\gamma }} \biggr] \frac{\widetilde{V}_{n}^{(1-\sigma)(q-1)}\mu(x)}{U^{1-\delta\sigma }(x)\nu_{n}^{q-1}}a_{n}^{q}\,dx \Biggr\} ^{\frac{1}{q}} \\ =&\bigl(k(\sigma)\bigr)^{\frac{1}{p}} \Biggl[ \sum _{n=1}^{\infty}\varpi_{\delta }(\sigma,n) \frac{\widetilde{V}_{n}^{q(1-\sigma)-1}}{\nu_{n}^{q-1}}a_{n}^{q} \Biggr] ^{\frac{1}{q}}. \end{aligned}$$

Then by the note of Lemma 2, we have (48). By the reverse Hölder inequality (*cf.* [31]), we have

50 $$\begin{aligned} I =& \int_{0}^{\infty} \biggl[ \bigl(1-\theta_{\delta}( \sigma,x)\bigr)^{\frac {1}{p}}\frac{U^{\frac{1}{q}-\delta\sigma}(x)}{\mu^{\frac {1}{q}}(x)}f(x) \biggr] \\ &{} \times \Biggl\{ \frac{U^{\delta\sigma-\frac{1}{q}}(x)\mu^{\frac {1}{q}}(x)}{(1-\theta_{\delta}(\sigma,x))^{\frac{1}{p}}}\sum_{n=1}^{\infty} \ln \biggl[ 1+\frac{\rho}{(U^{\delta}(x)\widetilde{V}_{n})^{\gamma}} \biggr] a_{n} \Biggr\} \,dx\geq \Vert f \Vert _{p,\widetilde{\Phi }_{\delta}}J. \end{aligned}$$

Then by (48), we have (46). On the other hand, assuming that (46) is valid, we set $f(x)$ as in Theorem 1. Then we find $J^{q}= \Vert f \Vert _{p,\widetilde{\Phi}_{\delta}}^{p}$. If $J=\infty$, then (48) is trivially valid; if $J=0$, then (48) remains impossible. Suppose that $0< J<\infty$. By (46), it follows that

$$\Vert f \Vert _{p,\widetilde{\Phi}_{\delta }}^{p}=J^{q}=I>k(\sigma) \Vert f \Vert _{p,\widetilde{\Phi}_{\delta}} \Vert a \Vert _{q,\widetilde{\Psi }}, \quad\quad \Vert f \Vert _{p,\widetilde{\Phi}_{\delta}}^{p-1}=J>k(\sigma) \Vert a \Vert _{q,\widetilde{\Psi}}, $$

and then (48) follows, which is equivalent to (46).

Therefore, inequalities (46), (47) and (48) are equivalent.

For $\varepsilon\in(0,p\sigma)$, we set $\widetilde{\sigma}=\sigma+ \frac{\varepsilon}{p}$, and $\widetilde{f}=\widetilde{f}(x)$, $x\in \mathbf{R}_{+}$, $\widetilde{a}=\{\widetilde{a}_{n}\}_{n=1}^{\infty}$,

$$\begin{aligned}& \widetilde{f}(x) = \textstyle\begin{cases} U^{\delta\widetilde{\sigma}-1}(x)\mu(x),&0< x^{\delta}\leq1, \\ 0,&x^{\delta}>0,\end{cases}\displaystyle \\& \widetilde{a}_{n} =\widetilde{V}_{n}^{\widetilde{\sigma}-\varepsilon -1} \nu_{n}=\widetilde{V}_{n}^{\sigma-\frac{\varepsilon}{q}-1}\nu _{n},\quad n \in\mathbf{N}. \end{aligned}$$

By (19), (31) and the note of Lemma 2, we obtain

$$\begin{aligned}& \begin{aligned} \Vert \widetilde{f} \Vert _{p,\widetilde{\Phi}_{\delta }} \Vert \widetilde{a} \Vert _{q,\widetilde{\Psi}}&= \biggl[ \int_{\{x>0;0< x^{\delta}\leq1\} }\bigl(1-O\bigl(\bigl(U(x)\bigr)^{\frac{\delta\sigma}{2}}\bigr) \bigr)\frac{\mu(x)\,dx}{U^{1-\delta\varepsilon }(x)} \biggr] ^{\frac{1}{p}} \\ &\quad{} \times \Biggl( \sum_{n=1}^{\infty} \frac{\nu_{n}}{\widetilde{V}_{n}^{1+\varepsilon}} \Biggr) ^{\frac{1}{q}}=\frac{1}{\varepsilon} \bigl( U^{\delta\varepsilon}(1)-\varepsilon O_{1}(1) \bigr) ^{\frac {1}{p}} \biggl( \frac{1}{\widetilde{V}_{n_{0}}^{\varepsilon}}+\varepsilon O(1) \biggr) ^{\frac{1}{q}}, \end{aligned} \\& \widetilde{I} = \sum_{n=1}^{\infty} \int_{0}^{\infty}\ln \biggl[ 1+\frac{\rho}{(U^{\delta}(x)\widetilde{V}_{n})^{\gamma}} \biggr] \widetilde {a}_{n}\widetilde{f}(x)\,dx \\& \hphantom{\widetilde{I}} = \sum_{n=1}^{\infty} \biggl\{ \int_{\{x>0;0< x^{\delta}\leq1\}}\ln \biggl[ 1+\frac{\rho}{(U^{\delta}(x)\widetilde{V}_{n})^{\gamma}} \biggr] \frac {\widetilde{V}_{n}^{\widetilde{\sigma}}\mu(x)}{U^{1-\delta\widetilde{\sigma}}(x)}\,dx \biggr\} \frac{\nu_{n}}{\widetilde {V}_{n}^{1+\varepsilon}} \\& \hphantom{\widetilde{I}} \leq \sum_{n=1}^{\infty} \biggl\{ \int_{0}^{\infty}\ln \biggl[ 1+\frac {\rho }{(U^{\delta}(x)\widetilde{V}_{n})^{\gamma}} \biggr] \frac{\widetilde {V}_{n}^{\widetilde{\sigma}}\mu(x)}{U^{1-\delta\widetilde{\sigma}}(x)}\,dx \biggr\} \frac{\nu_{n}}{\widetilde{V}_{n}^{1+\varepsilon}} \\& \hphantom{\widetilde{I}} = \sum_{n=1}^{\infty}\varpi_{\delta}( \widetilde{\sigma},n)\frac {\nu_{n}}{\widetilde{V}_{n}^{1+\varepsilon}}=k(\widetilde{\sigma})\sum _{n=1}^{\infty}\frac{\nu_{n}}{\widetilde{V}_{n}^{1+\varepsilon }} \\& \hphantom{\widetilde{I}} = \frac{1}{\varepsilon}k\biggl(\sigma+\frac{\varepsilon}{p}\biggr) \biggl( \frac {1}{\widetilde{V}_{n_{0}}^{\varepsilon}}+\varepsilon O(1) \biggr) . \end{aligned}$$

If there exists a positive constant $K\geq k(\sigma)$, such that (41) is valid when replacing $k(\sigma)$ to *K*, then in particular we have $\varepsilon\widetilde{I}>\varepsilon K \Vert \widetilde{f} \Vert _{p,\widetilde{\Phi}_{\delta}} \Vert \widetilde{a} \Vert _{q,\widetilde{\Psi}}$, namely,

$$k\biggl(\sigma+\frac{\varepsilon}{p}\biggr) \biggl( \frac{1}{\widetilde{V}_{n_{0}}^{\varepsilon}}+ \varepsilon O(1) \biggr) >K \bigl( U^{\delta \varepsilon}(1)-\varepsilon O_{1}(1) \bigr) ^{\frac{1}{p}} \biggl( \frac {1}{\widetilde{V}_{n_{0}}^{\varepsilon}}+ \varepsilon O(1) \biggr) ^{\frac{1}{q}}. $$

It follows that $k(\sigma)\geq K$ ($\varepsilon\rightarrow0^{+}$). Hence, $K=k(\sigma)$ is the best possible constant factor of (46). The constant factor $k(\sigma)$ in (47) ((48)) is still the best possible. Otherwise, we would reach the contradiction by (49) ((50)) that the constant factor in (46) is not the best possible. The theorem is proved. □

### Remark

(i) For $\delta=-1$ in (20), we obtain the following inequality with the homogeneous kernel of degree 0:

51 $$ \sum_{n=1}^{\infty} \int_{0}^{\infty}\ln \biggl[ 1+\rho \biggl( \frac {U(x)}{\widetilde{V}_{n}} \biggr) ^{\gamma} \biggr] a_{n}f(x)\,dx< \frac{\rho ^{\sigma /\gamma}\pi}{\sigma\sin(\frac{\pi\sigma}{\gamma})} \Vert f \Vert _{p,\Phi _{-1}} \Vert a \Vert _{q,\widetilde{\Psi}}. $$

(ii) For $\delta=1$ in (20), we obtain the following inequality with the non-homogeneous kernel:

52 $$ \sum_{n=1}^{\infty} \int_{0}^{\infty}\ln \biggl[ 1+\frac{\rho}{(U(x)\widetilde{V}_{n})^{\gamma}} \biggr] a_{n}f(x)\,dx< \frac{\rho^{\sigma /\gamma }\pi}{\sigma\sin(\frac{\pi\sigma}{\gamma})} \Vert f \Vert _{p,\Phi_{1}} \Vert a \Vert _{q,\widetilde{\Psi}} . $$

(iii) For $\widetilde{\mu}_{n}=0$ ($n\in\mathbf{N}$) in (20), we have the following inequality:

53 $$ \sum_{n=1}^{\infty} \int_{0}^{\infty}\ln \biggl[ 1+\frac{\rho }{(U^{\delta }(x)V_{n})^{\gamma}} \biggr] a_{n}f(x)\,dx< \frac{\rho^{\sigma/\gamma }\pi}{\sigma\sin(\frac{\pi\sigma}{\gamma})} \Vert f \Vert _{p,\Phi_{\delta }} \Vert a \Vert _{q,\Psi}, $$

where the constant factor $\frac{\rho^{\sigma/\gamma}\pi}{\sigma \sin(\frac{\pi\sigma}{\gamma})}$ is still the best possible. Hence, inequality (20) is a more accurate form of (53) (for $0<\widetilde {\mu}_{n}\leq\frac{\mu_{n}}{2}$, $n\in\mathbf{N}$).

(iv) For $\mu(x)=\mu_{n}=1$ ($x\in\mathbf{R}_{+}$, $n\in\mathbf{N}$), $\delta=-1$ in (53), we have the following inequality:

54 $$\begin{aligned}& \sum_{n=1}^{\infty}a_{n} \int_{0}^{\infty}\ln \biggl[ 1+\rho \biggl( \frac{x}{n} \biggr) ^{\gamma} \biggr] f(x)\,dx \\& \quad = \int_{0}^{\infty}f(x)\sum_{n=1}^{\infty} \ln \biggl[ 1+\rho \biggl( \frac{x}{n} \biggr) ^{\gamma} \biggr] a_{n}\,dx \\& \quad < \frac{\rho^{\sigma/\gamma}\pi}{\sigma\sin(\frac{\pi\sigma }{\gamma })} \biggl[ \int_{0}^{\infty}x^{p(1+\sigma)-1}f^{p}(x)\,dx \biggr]^{\frac {1}{p}} \Biggl[\sum_{n=1}^{\infty}n^{q(1-\sigma)-1}b_{n}^{q} \Biggr]^{\frac{1}{q}}, \end{aligned}$$

which is a particular case of (8) for $\lambda=0$, $\lambda _{1}=-\sigma$, $\lambda_{2}=\sigma$ and $k_{\lambda}(x,n)=\ln [ 1+\rho ( \frac{x}{n} ) ^{\gamma} ] $.

We still can obtain some inequalities with the best possible constant factors in Theorems 1-4, by using some particular parameters.

## Conclusions

In this paper, by means of the weight functions, the technique of real analysis and Hermite-Hadamard’s inequality, a more accurate half-discrete Hardy-Hilbert-type inequality related to the kernel of logarithmic function and a best possible constant factor is given by Theorems 1-2. Moreover, the equivalent forms and the operator expressions are considered. We also obtain the reverses and some particular cases in Theorems 3-4. The method of weight functions is very important, which is the key to help us proving the main inequalities with the best possible constant factor. The lemmas and theorems provide an extensive account of this type of inequalities.
